# Serum IL-6 and functional outcome after endovascular therapy: the mediating role of infarct volume growth in acute ischemic stroke

**DOI:** 10.3389/fimmu.2026.1800891

**Published:** 2026-08-10

**Authors:** Shuo Li, Shiyao Zhang, Lifan Ji, Yanfang Qu, Zhihui Huang, Jie Gao, Yiting Zhang, Zhenzhen Li, Huiling Sun, Lin Zhu, Teng Jiang, Yadong Yu, Qiwen Deng

**Affiliations:** 1 Department of Neurology, Nanjing First Hospital, Nanjing Medical University, Nanjing, China; 2 Department of Neurology, Affiliated ZhongDa Hospital, School of Medicine, Southeast University, Nanjing, China; 3 School of Basic Medicine and Clinical Pharmacy, China Pharmaceutical University, Nanjing, China; 4 General Clinical Research Center, Nanjing First Hospital, Nanjing Medical University, Nanjing, China; 5 Department of Neurology, Lianshui County People’s Hospital, Huai’an, China

**Keywords:** acute ischemic stroke, endovascular therapy, IL-6, infarct volume growth, inflammatory factors

## Abstract

**Background and purpose:**

Inflammatory factors are pivotal in systemic and central nervous system inflammation post-stroke. This study examined if peripheral inflammatory factors were associated with outcomes after endovascular therapy for acute large vessel occlusion stroke by mediating infarct growth.

**Methods:**

We conducted a retrospective analysis of the EVTRNA (Circulating Non-coding RNA in Acute Ischemic Stroke with Endovascular Treatment) trial. Patients who underwent pre-endovascular therapy multimodal MRI, postoperative day 1 inflammatory factor testing, and postoperative day 7 head CT for infarct volume evaluation were included in this study. Unfavorable outcome was defined as a modified Rankin Scale score of 3–5 at the 90-day follow-up. Regression analysis and mediation analysis were employed to assess the association between inflammatory factors and clinical outcomes.

**Results:**

A total of 105 patients were included in this study, of whom 51 experienced unfavorable outcomes. Univariate analysis demonstrated that elevated interleukin-6 (IL-6) levels, higher baseline National Institutes of Health Stroke Scale scores at admission, increased fasting blood glucose levels, and infarct growth were all significantly associated with unfavorable outcomes. After adjustment by multivariate analysis, IL-6 (odds ratio 1.02, 95% confidence interval 1.01-1.03) and the other aforementioned factors remained independently associated with unfavorable outcomes. Further mediation analysis revealed that infarct volume growth partially accounts for the unfavorable prognosis observed in patients with elevated IL-6 levels.

**Conclusions:**

IL-6 was associated with unfavorable outcomes in patients undergoing endovascular therapy. Postoperative infarct volume growth partially mediated the effect of IL-6 on clinical outcomes.

## Introduction

As the gold standard treatment for acute large vessel occlusion (LVO) stroke, endovascular therapy (EVT) has significantly improved reperfusion rates (1, 2). Nevertheless, unfavorable clinical outcomes persist in approximately 54% of patients (3), which may be mediated by thrombosis and inflammatory responses (4). Inflammatory factors are a key mediator in this reperfusion injury (5, 6). Accumulating observational evidences have indicated that inflammatory factors serve as predictors for unfavorable outcomes in patients undergoing EVT, including futile recanalization (7) and early neurological deterioration (8). However, the clinical mechanisms linking inflammatory factors to unfavorable outcome in EVT patients have not been elucidated.

Previous studies have demonstrated that the involvement of inflammatory factors in reperfusion injury pathways can alter infarct volume (5), ultimately making it a key determinant of functional outcome based on modified Rankin Scale (mRS) assessment (9, 10). Critically, infarct volume growth, the radiological evolution of tissue injury from baseline to follow-up imaging, has been established as a determinant of clinical outcome in LVO patients treated with EVT (11, 12). Whether inflammatory factors influence clinical outcomes by modulating infarct volume growth in EVT patients remains unknown.

We analyzed data from the EVTRNA trial (Circulating Non-coding RNA in Acute Ischemic Stroke with Endovascular Treatment) to assess whether inflammatory factors within 24 h and infarct volume growth are associated with 90-day functional outcomes after EVT in acute ischemic stroke (AIS) patients due to LVO. Additionally, we used mediation analysis to examine the potential role of infarct volume growth as a mediator between inflammatory factors and clinical prognosis.

## Methods

### Study population

EVTRNA trial was a cohort study starting in March 2020 that included acute LVO patients treated with EVT in Nanjing First Hospital, Nanjing Medical University (URL: www.clinicaltrials.gov, unique identifier: NCT04230785). We included AIS patients with anterior circulation occlusion were first assessed with pre-EVT multimodal MRI for baseline infarct volume, subsequently underwent inflammatory factor testing on postoperative within 24 h, and finally received a head CT on postoperative day 7 for final infarct volume evaluation. Patients met any of the following exclusion criteria were excluded: (1) Patients with a history of severe or active infection within the 6 months prior to enrollment; (2) patients who had received antibiotic therapy, psychotropic medications, immunosuppressive therapy, or had undergone radiotherapy/chemotherapy within the preceding 6 months; (3) patients who under 18 years of age; (4) patients who have a malignant tumor, an autoimmune disease, or dysfunction of vital organs (e.g., heart, liver, kidneys); (5) Patients who died within 7 days following disease onset. A total of 105 AIS patients due to LVO were included to investigate the mediation effect of infarct volume growth between inflammatory factors and 90-day clinical outcome. The clinical baseline characteristics of patients are summarized in **Table 1**. Participants were followed up at 90-day through structured telephone interviews or outpatient clinic appointments. Favorable outcome was defined as a mRS score of 0–2 at the 90-day follow-up, and mRS score of 3–5 was considered as unfavorable outcome. The study was approved by the Nanjing Medical University Ethics Committee and each patient or their legally authorized representative provided written informed consent.

**Table 1:** Baseline characteristics of eligible patients stratified by outcome.

Variable	Favorable outcome^#^ (n=54)	Unfavorable outcome^#^ (n=51)	*P*
Demographic characteristics, mean (SD), or n (%)			
Age,	68.09 (11.84)	71.61 (9.88)	0.103
Male	41 (75.93)	33 (64.71)	0.208
Vascular risk factors, n (%)			
Hypertension	32 (59.26)	31 (60.78)	0.873
Diabetes mellitus	10 (18.52)	14 (27.45)	0.276
Previous stroke	11 (20.37)	8 (15.69)	0.533
Atrial fibrillation	11 (20.37)	13(25.49)	0.532
Valvular heart disease	3 (5.56)	2 (3.92)	0.694
CAD	5 (9.26)	6 (11.76)	0.675
Drinking history	15 (27.78)	13 (25.49)	0.791
Smoking history	18 (33.33)	12 (23.53)	0.266
Heart failure	1 (1.85)	3 (5.88)	0.281
Cerebral hemorrhage	2 (3.70)	1 (1.96)	0.592
**TOAST classification, n (%)**			0.119
LA	28 (51.85)	21 (41.18)	
CE	20 (37.04)	28 (54.90)	
Others^*^	6 (11.11)	2 (3.92)	
**Occlusion location, n (%)**			0.842
ICA	12 (22.22)	9 (17.65)	
MCA	38 (70.37)	38 (74.51)	
TO	4 (7.41)	4 (7.84)	
**mTICI scale, n (%)**			0.052
2a	0 (0.00)	2 (3.92)	
2b	16 (29.63)	22 (43.14)	
3	38 (70.37)	27 (52.94)	
Clinical assessment and treatment, median (IQR), mean (SD), or n (%)			
DPT, minutes	107.50 (84.75, 146.25)	100.00 (75.00, 130.00)	0.362
DRT, minutes	164.00 (140.25, 226.25)	195.00 (145.00, 242.00)	0.383
Within 4.5-hour time window^&^	26 (48.15)	24 (47.06)	0.911
mRS on admission	0 (0,0)	0 (0,0)	0.187
NIHSS on admission	12.30 (4.42)	15.00 (4.85)	**0.004**
ASPECT score before EVT	9 (8, 10)	9 (8, 10)	0.205
Intravenous thrombolysis	21 (38.89)	16 (31.37)	0.420
Baseline infarct volume (ml)	2.12 (0.00, 11.58)	3.01 (0.00, 25.0)	0.322
FIV (ml)	16.01 (3.88, 34.55)	58.89 (15.94, 159.93)	**<0.001**
Infarct Volume Growth (ml)	6.73 (-0.16, 27.11)	46.49 (5.51, 135.47)	**<0.001**
Hemorrhagic transformation	9 (16.67)	14 (27.45)	0.182
Laboratory parameters (mmol/l), median (IQR), or mean (SD)			
Fasting Blood Glucose	5.60 (4.88, 7.19)	7.23 (5.72, 8.61)	**0.001**
TG	1.12 (0.80, 1.50)	0.94 (0.79, 1.30)	0.238
LDL-C	2.61 (0.93)	2.41 (0.76)	0.213
HDL-C	1.15 (0.30)	1.11 (0.36)	0.565
Inflammatory factor (pg/mL), median (IQR), or mean (SD)			
TNF-α	1.73 (0.88, 2.82)	1.65 (0.98, 3.03)	0.736
IFN-γ	2.46 (1.80, 3.53)	2.15 (1.53, 3.48)	0.403
IL-10	4.25 (3.04, 5.80)	5.14 (3.67, 7.03)	0.065
IL-2	3.09 (2.23, 3.57)	2.65 (1.98, 3.82)	0.507
IL-4	2.21 (1.16)	2.62 (1.45)	0.118
IL-6	15.68 (9.35, 34.27)	24.70 (14.32, 56.20)	**0.004**

Standard deviation, SD; Interquartile range, IQR; CAD, Coronary Artery Disease; TOAST, Trial of Org 10172 in Acute Stroke Treatment; mTICI, modified Thrombolysis in Cerebral Infarction scale; LA, large-artery atherosclerosis; CE, cardio-embolism; ICA, internal carotid artery; MCA, middle cerebral artery; TO, tandem occlusions; DPT, door-to-puncture time; DRT, door-to-reperfusion time; ASPECT, Alberta Stroke Program Early Computed Tomography Score. The 4.5-hour time window, The 4.5-hour thrombolysis time window; NIHSS, National Institutes of Health Stroke Scale; mRS, Modified Rankin Scale; TG, triglycerides; LDL-C, low-density lipoprotein cholesterol; HDL-C, high-density lipoprotein cholesterol; FIV, final infarct volumssadfe; TNF-α, tumor necrosis factor-α; IFN-γ, interferon-γ; IL, interleukin.

^*^Others include small-vessel occlusion, stroke of other determined etiology and undetermined etiology.

^#^Favorable outcome was defined as a mRS score of 0–2 at the 90-day follow-up, and mRS score of 3–5 was considered as Unfavorable outcome.

^&^The 4.5-hour time window refers to onset-to-arrival time ≤4.5 h at our center, several patients presented beyond this window yet received pre-transfer thrombolysis at outside hospitals.Bold values indicate statistically significant differences between groups.

### Neuroimaging

**Figure 1:**
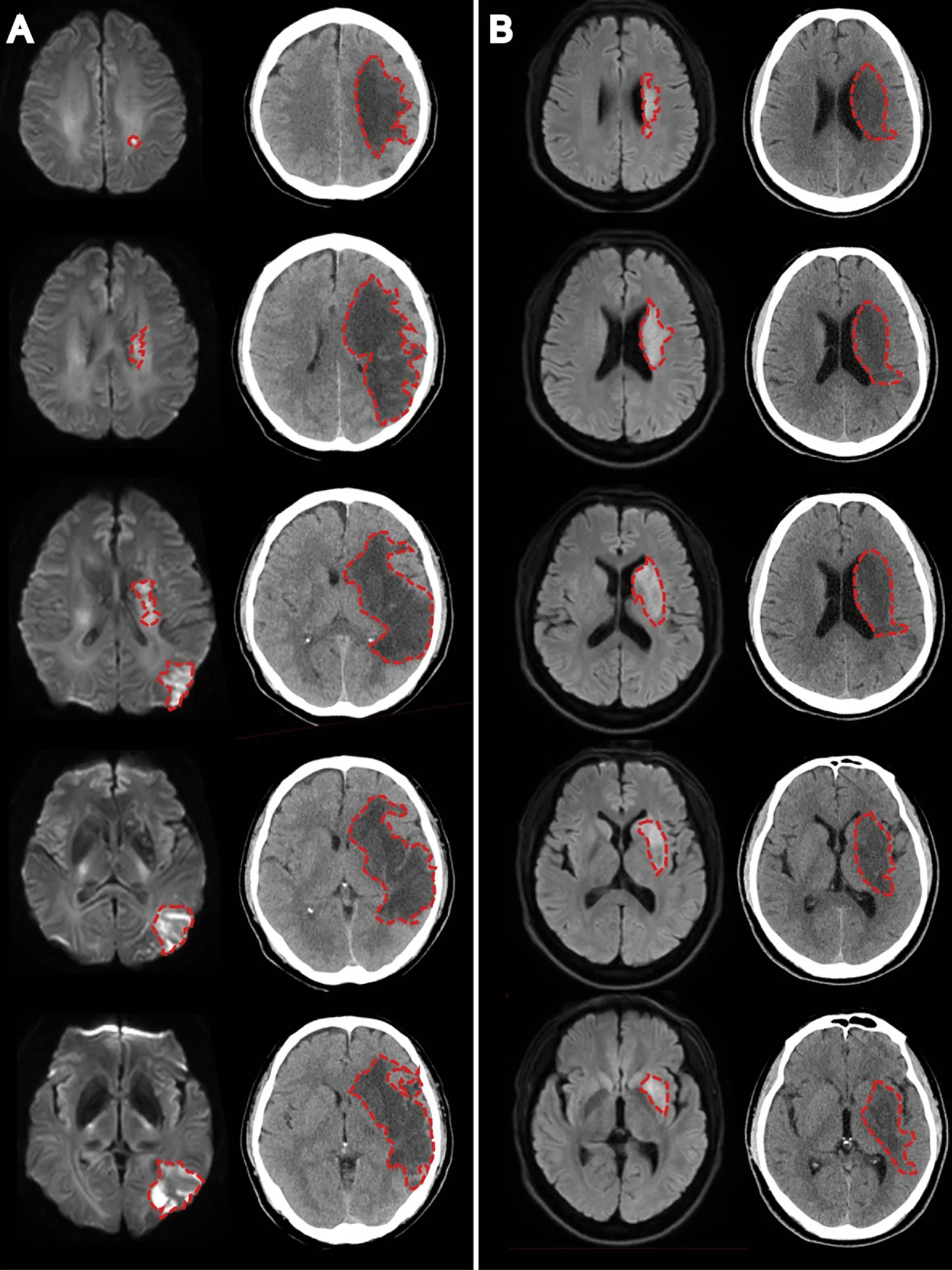
Schematic diagram of semi-quantitative infarct volume calculation. Infarct regions were delineated as hyper intense areas on pre-treatment MRI-DWI sequences and as hypodense areas on post-treatment CT scans (red outlines). **(A)** Unfavorable outcome group: CT imaging revealed significant cerebral edema, necessitating correction. The infarct volume was calculated as hypodense volume on CT- (area of the ipsilateral hemisphere - area of the contralateral hemisphere). **(B)** Favorable outcome group: For each imaging slice, the lesion area was calculated and multiplied by the slice thickness to derive the lesion volume. Infarct volume growth (ΔIV) was quantified as the difference between the final and baseline volumes. MRI, magnetic resonance imaging; DWI, diffusion-weighted imaging; CT, computed tomography.

Multimodal MRI was performed in all participants prior to EVT, with baseline infarct volume measured on diffusion-weighted imaging (DWI) sequences. A follow-up computed tomography (CT) scan was conducted at day 7 for calculation of the final infarct volume. All volume assessments were carried out by stroke neurologists with subspecialty expertise in neuroradiology blinded to clinical and follow-up data. Infarct volumes were measured using automated volumetric calculation by applying a standardized apparent diffusion coefficient (ADC) cutoff value of ADC < 620×10^-6^mm²/s with F-STROKE software (Institute of Science and Technology for Brain-Inspired Intelligence, Fudan University and Neuroblem (Shanghai) Intelligent Technology Co., Ltd., Shanghai, China) or a semi-automated process, which involved tracing the boundaries of corresponding high- and low-attenuation or signal intensity zones on each slice image (**Figure 1**). If the infarct lesion exhibited edema, the contralateral brain area was further outlined for correction, where the infarct volume = (hypodense volume in CT or hype-attenuation volume in DWI sequences) - (area of the ipsilateral hemisphere - area of the contralateral hemisphere). The lesion area per slice was then calculated and multiplied by the slice thickness to yield the lesion volume. Infarct volume growth (delta IV, ΔIV) was quantified by subtracting the baseline volume from the final volume.

### Blood sampling

Blood samples were collected within 24 hours after EVT, considered as day 1. Serum was separated by centrifugation at 1500rpm for 15 minutes at 4 °C and stored at –80 °C until analysis. The serum concentrations of interleukin (IL)-2, IL-4, IL-6, IL-10, tumor necrosis factor-alpha (TNF-α), and interferon-gamma (IFN-γ) were quantified using the Navios flow cytometer (Beckman Coulter, California, USA).

### Statistical analysis

Categorical variables were presented as frequencies with percentages. Continuous variables were described using median and interquartile range (IQR) for non-normally distributed data, and mean ± standard deviation (SD) for normally distributed data. Group comparisons for continuous variables were performed using the independent samples t-test, the Mann-Whitney U-test or Kruskal-Wallis test, as appropriate. Categorical variables were compared using Fisher’s exact test or Pearson’s chi-squared test. A two-sided *P* value < 0.05 was considered statistically significant. Logistic regression analysis was used to examine the associations of IL-6 and infarct volume growth with prognosis. Variables that were significant in univariate logistic regression were entered into the multivariate analysis. Spearman correlation analysis was used to test the association between IL6 and infarct volume growth. Mediation analysis was performed to assess the potential mediating role of infarct volume growth in the association between IL-6 and clinical prognosis. Variables showing significant associations in multivariable logistic regression analysis (*P* value < 0.05) were included as covariates to adjust the mediation effect model. All statistical analyses were performed using SPSS 32.0.0.0 (IBM, Armonk, NY, USA), and graphs were generated with GraphPad Prism 10.0.

## Results

### Study population

**Figure 2:**
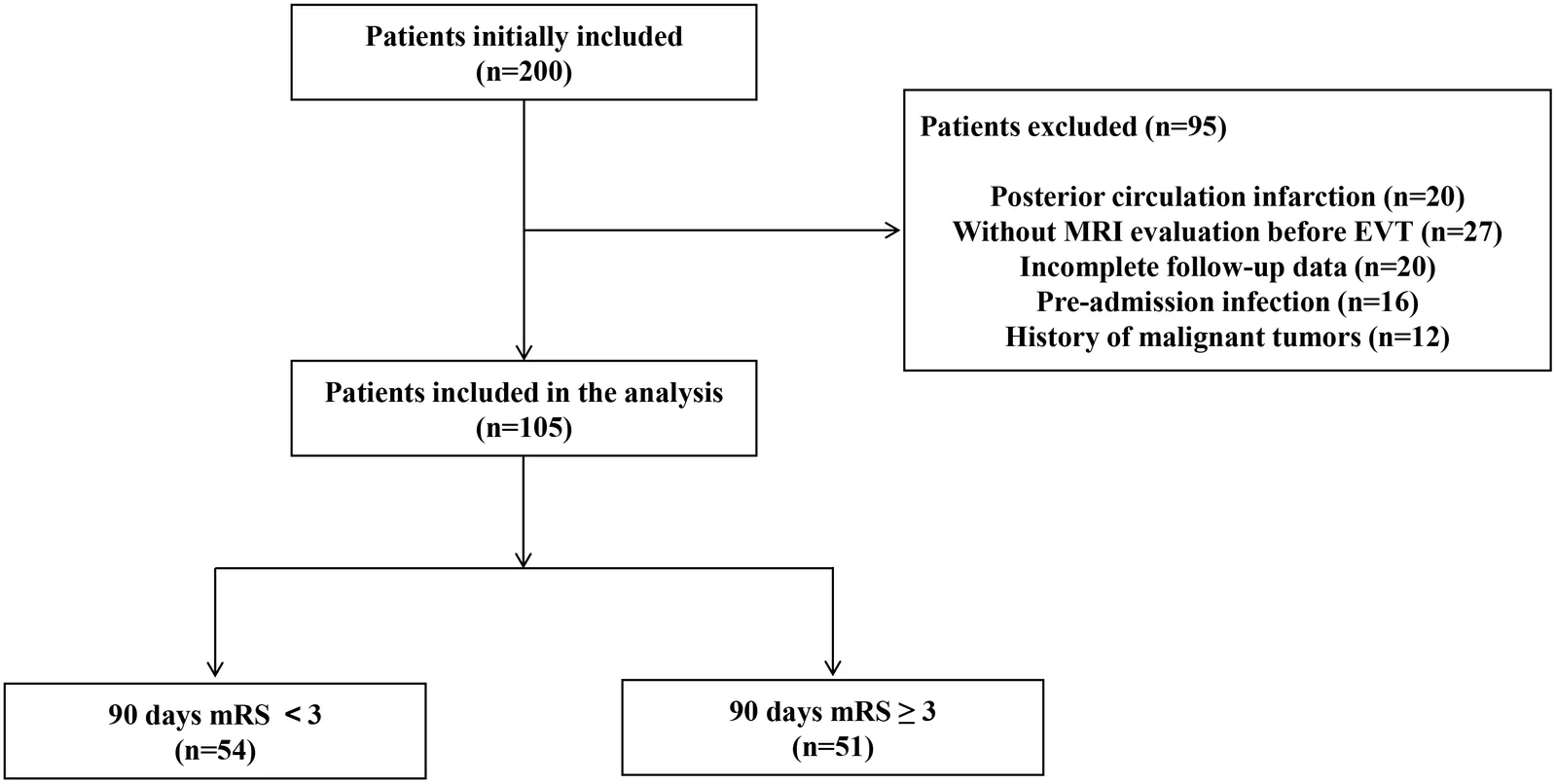
The flowchart of subject enrollment. MRI, magnetic resonance imaging. mRS, modified rankin scale.

Of 200 patients enrolled in the EVTRNA trial between September 2020 and June 2022, 105 patients (male sex, n=74 (70%); mean age, 69.8 ± 11.02 years) met the inclusion criteria and were included in the study (**Figure 2**). Patients (n=95) were excluded due to posterior circulation infarction, without MRI evaluation before EVT, incomplete follow-up data, infection before admission and history of malignant tumors. Of 105 patients included in the study, 54 patients (51.4%) achieved favorable outcomes, while unfavorable outcomes were observed in 51 patients (48.6%). The clinical baseline characteristics of two group patients are summarized in **Table 1**. Variables such as age, sex, vascular risk factors (including hypertension, diabetes, atrial fibrillation, and history of smoking or alcohol), TOAST classification, occlusion location, modified Thrombolysis in Cerebral Infarction (mTICI) scale, door-to-puncture time (DPT), door-to-recanalization time (DRT), Alberta Stroke Program Early Computed Tomography (ASPECT) Score, the proportion receiving intravenous thrombolysis, baseline infarct volume, the proportion of hemorrhagic transformation and serum lipid levels (triglycerides, low-density lipoprotein cholesterol (LDL-C), and high-density lipoprotein cholesterol (HDL-C)) did not differ significantly between the favorable and unfavorable prognosis groups (all *P >*0.05). However, significant differences were observed between the two groups in terms of the baseline National Institutes of Health Stroke Scale (NIHSS) score on admission (*P* = 0.004), fasting blood glucose (*P* = 0.001), and the final infarct volume (*P* < 0.001).

### Inflammatory factors and infarct volume growth by outcome group

**Figure 3:**
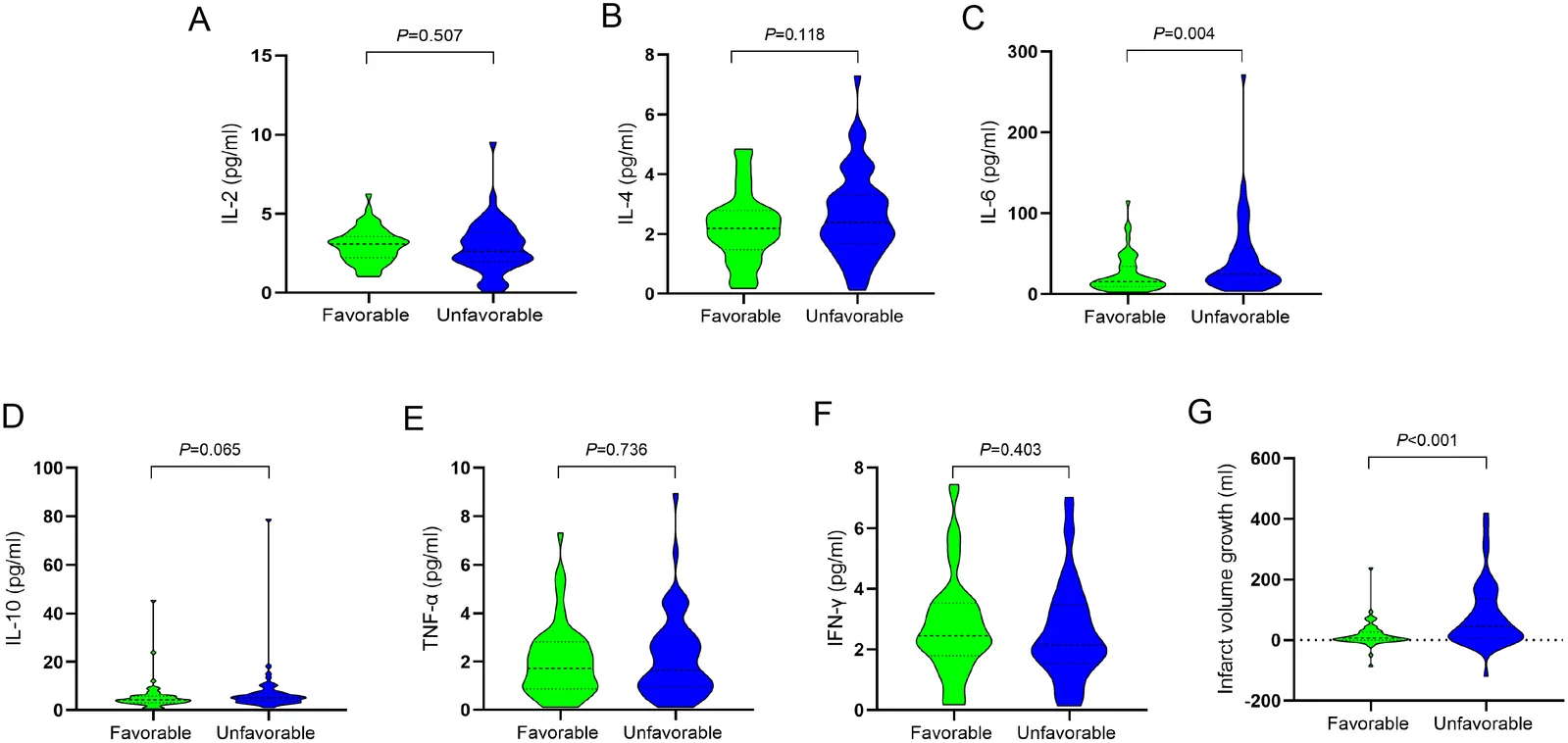
Expression of serum inflammatory factors (IL-2, IL-4, IL-6, IL-10, TNF-α, and IFN-γ) in the favorable versus unfavorable outcome groups (n=54 vs. n=51), as detected by Navios flow cytometry **(A–F)**. **(G)** shows the comparison of infarct volume growth between the two groups. IL, interleukin; TNF-α, tumor necrosis factor-alpha; IFN-γ, interferon-gamma.

Among multiple inflammatory factors (IL-2, IL-4, IL-6, IL-10, TNF-α and IFN-γ) analyzed, only IL-6 showed a significant difference between the favorable and unfavorable outcome groups (**Figures 3A-F**; **Table 1**). Patients with an unfavorable outcome had a higher median IL-6 level than those with a favorable outcome (15.68 pg/ml [IQR, 9.35-34.27] versus 24.70 pg/ml [IQR, 14.32-56.20], *P* = 0.004). Furthermore, the unfavorable outcome group exhibited greater infarct volume growth than the good outcome group (46.49 ml [IQR, 5.51-135.47] versus 6.73 ml [IQR, -0.16-27.11], *P* < 0.001) (**Figure 3G**; **Table 1**).

### Association of IL-6 and infarct volume growth with outcomes

**Figure 4:**
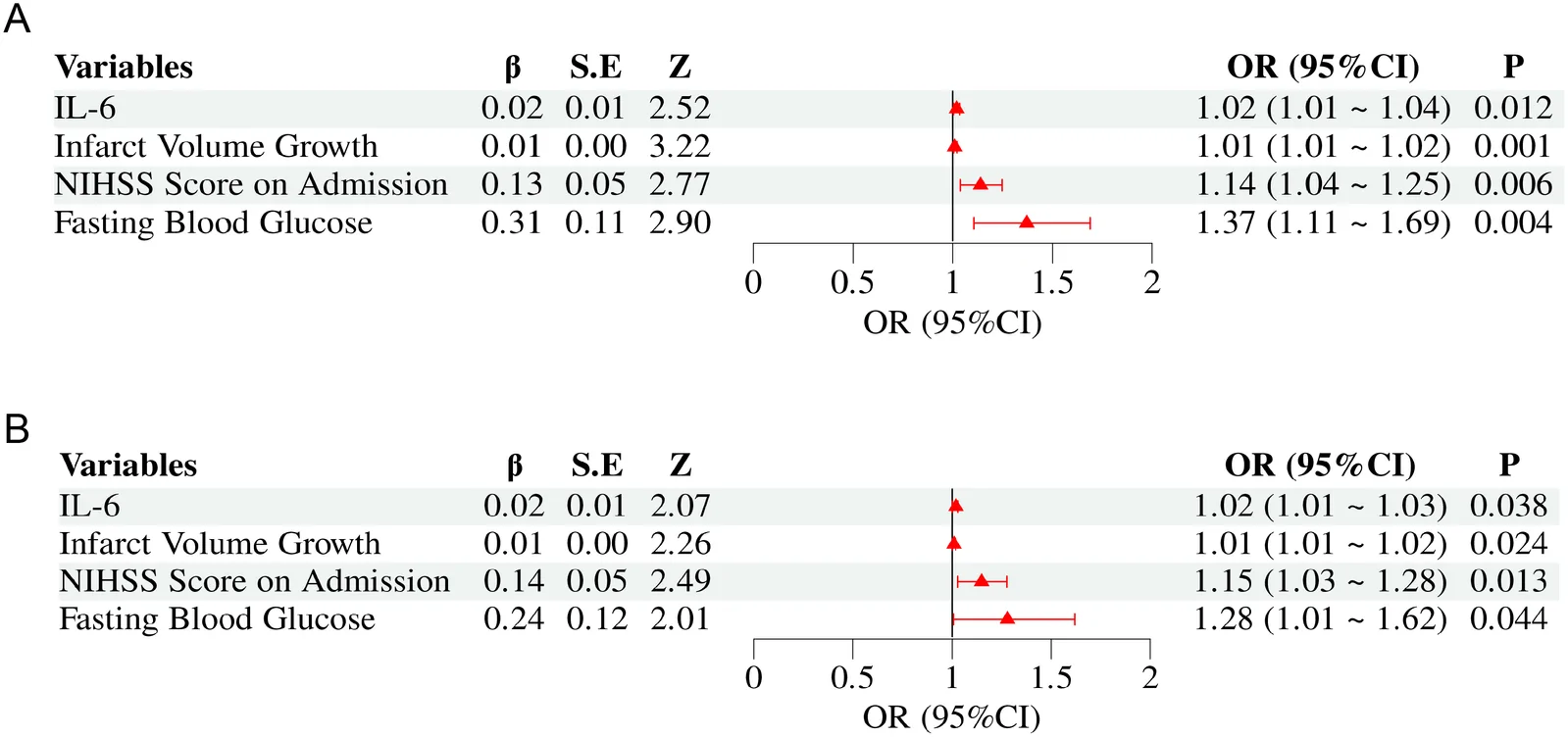
Univariate **(A)** and multivariate **(B)** logistic regression analysis for predicting unfavorable outcome.

To identify the predictors of clinical outcome, variables that showed significant differences between the favorable and unfavorable outcome groups were analyzed in the logistic regression model, including IL-6, NIHSS score on admission, infarct volume growth, and fasting blood glucose. Crude logistic regression analysis showed that IL-6 (odds ratio [OR], 1.02 [95% CI, 1.01–1.04], *P* = 0.012), NIHSS score on admission (OR, 1.14 [95% CI, 1.04–1.25], *P* = 0.006), infarct volume growth (OR, 1.01 [95% CI, 1.01–1.02], *P* = 0.001), and fasting blood glucose (OR, 1.37 [95% CI, 1.11–1.69], *P* = 0.004) were significantly associated with unfavorable outcome in stroke patients receiving EVT (**Figure 4A**). Furthermore, after adjusting for these potential confounders in the multivariable logistic regression model, a 1 pg/ml increase in IL-6 level was associated with a 2% increase in the odds of a unfavorable functional outcome (OR, 1.02 [95% CI, 1.01–1.03], *P* = 0.038). Consistent with this, infarct volume growth remained an independently associated with unfavorable outcome in the multivariable analysis (OR, 1.01 [95% CI, 1.01–1.02], *P* = 0.024), together with NIHSS score on admission and fasting blood glucose (**Figure 4B**).

### Relationship between IL-6, infarct volume growth and outcome

**Figure 5:**
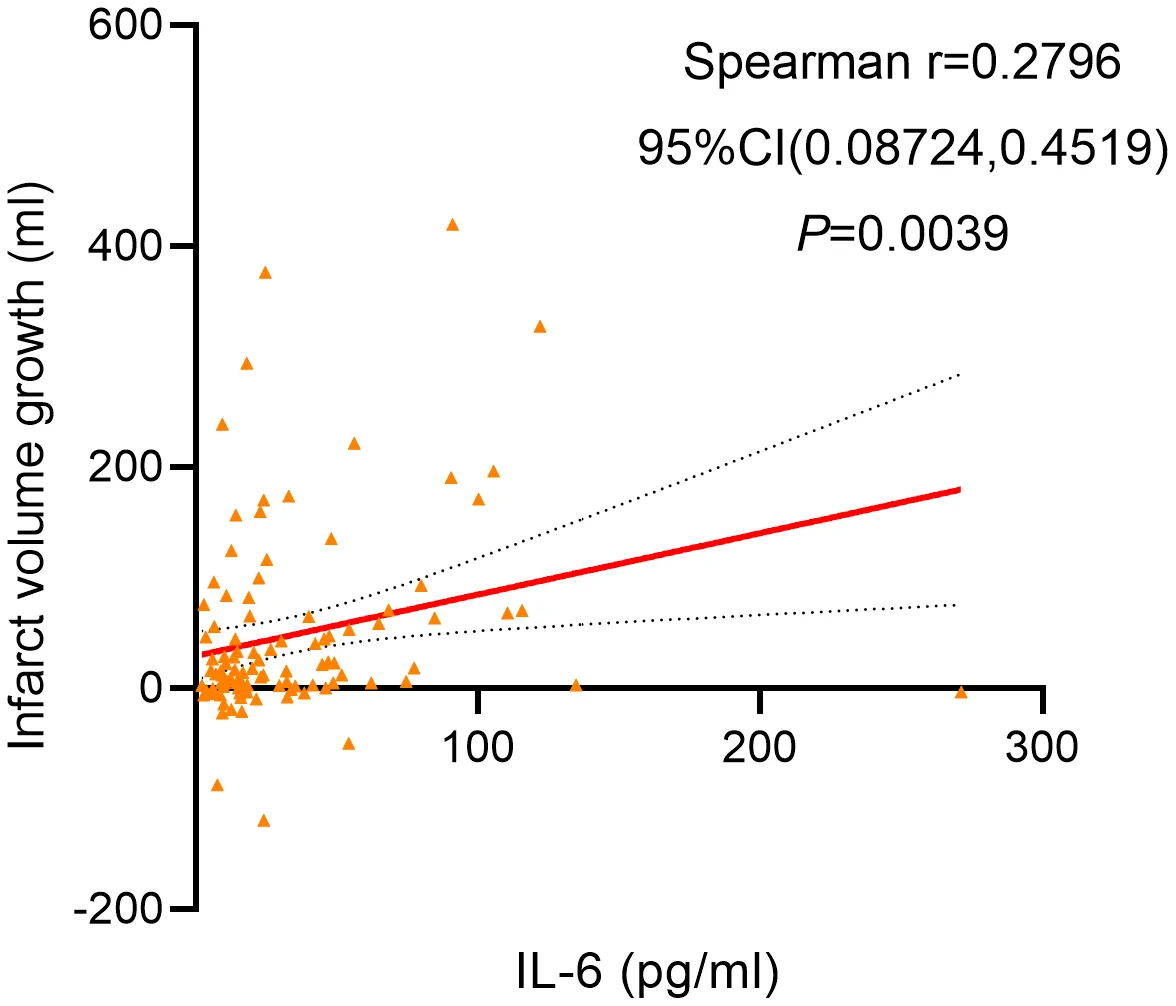
Spearman correlation analysis of IL-6 and infarct volume growth. IL-6, interleukin-6.

Based on previous evidence linking elevated serum IL-6 levels to larger infarct volumes in stroke patients (13), we proceeded to investigate the correlation between IL-6 and infarct volume growth. Spearman’s correlation analysis revealed a significant positive correlation between the two variables (r=0.2796, *P* = 0.0039), as detailed in **Figure 5**. Furthermore, previous studies have demonstrated that patients aged 85 years or more with AIS exhibited distinct risk factor profiles and prognostic characteristics (14). Accordingly, we performed a subgroup analysis focusing on this oldest-old population in the present study. The clinical baseline characteristics of eligible patients aged ≥85 years stratified by clinical outcome are summarized in the **Supplementary Table**. Inflammatory factors (IL-2, IL-4, IL-6, IL-10, TNF-α and IFN-γ) and infarct volume growth showed no significant differences between the favorable and unfavorable outcome groups among patients older than 85 years. However, IL-6 was markedly positively correlated with infarct volume growth (r=0.6485, *P* = 0.0490)(**Supplementary Figure 1**).

**Figure 6:**
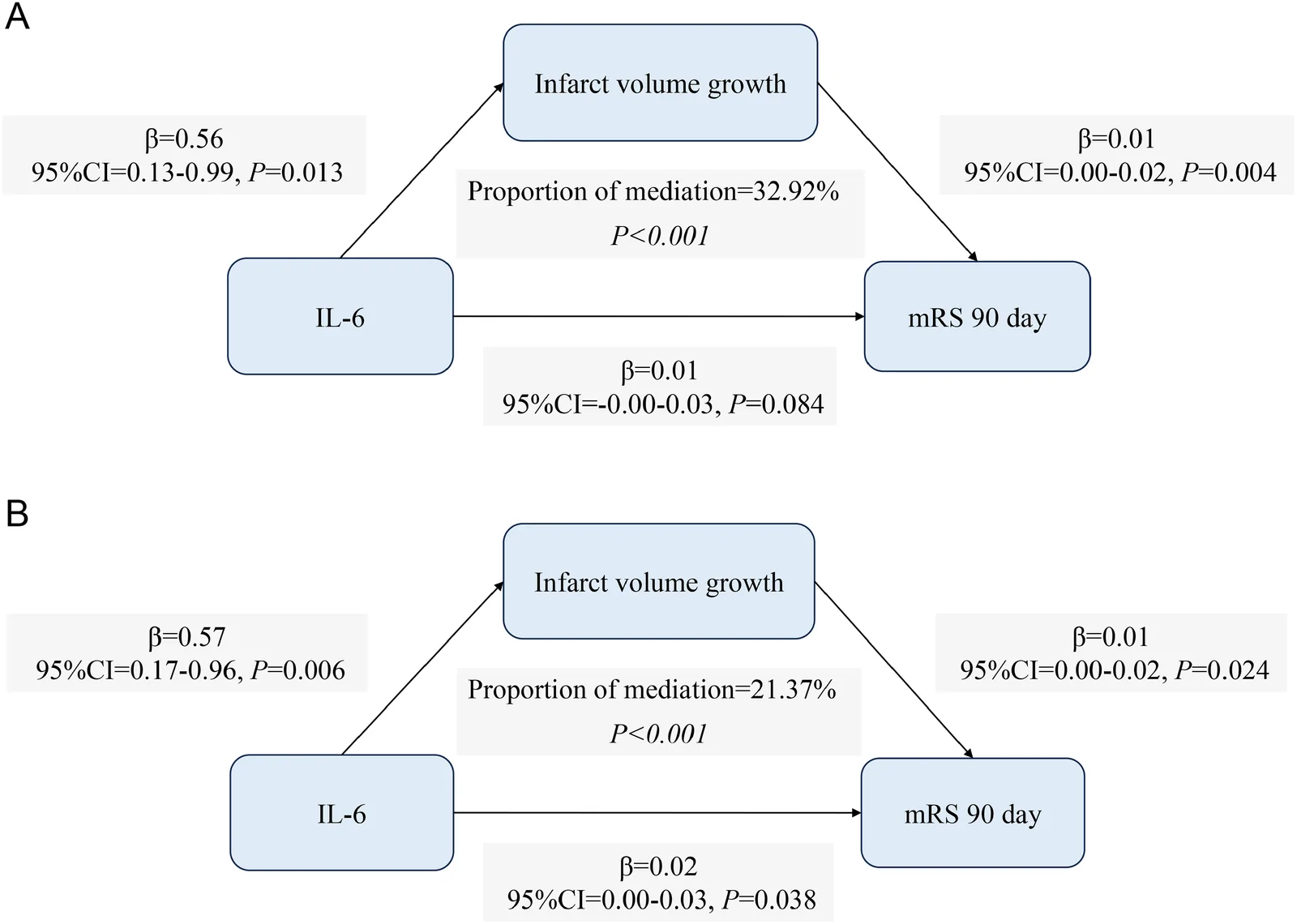
Analysis of the mediating effect of infarct volume growth on the relationship between IL-6 and functional outcome. **(A)** Mediation effect without covariates included. **(B)** Mediation effect with adjusting for baseline NIHSS score and fasting glucose. IL-6, interleukin-6.

To investigate whether infarct volume growth mediates the effect of IL-6 on unfavorable outcome, we performed a mediation analysis. As shown in **Figure 6A**, the mediation analysis demonstrated complete mediation of infarct volume growth in the absence of covariate adjustment. After adjusting for baseline NIHSS score and fasting glucose, mediation analysis confirmed a significant indirect effect of IL-6 on unfavorable outcome through infarct volume growth, accounting for 21.37% of the total effect (*P* < 0.001) (**Figure 6B**).

## Discussion

**Figure 7:**
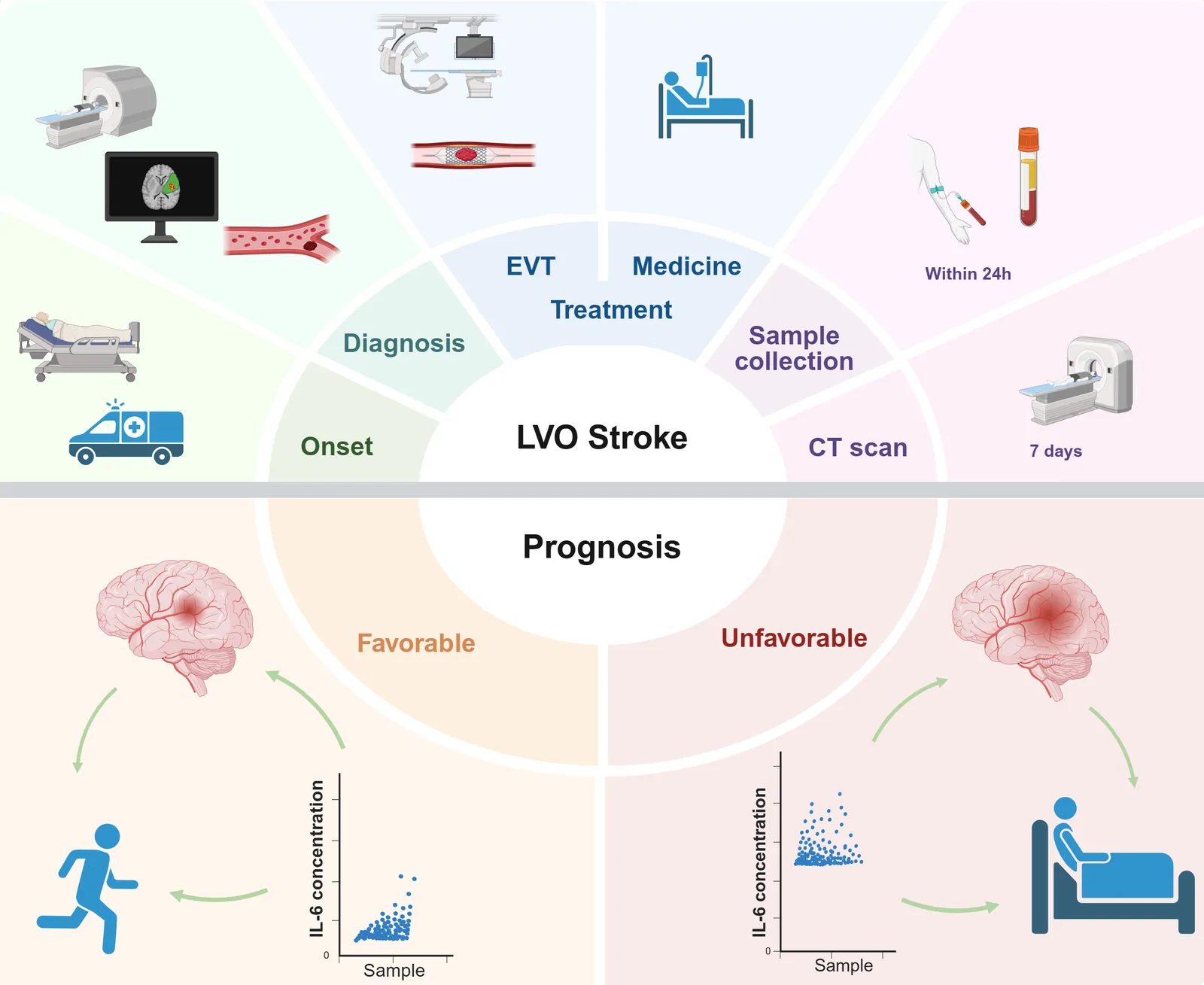
Graphic abstract. Higher levels of IL-6 contribute to unfavorable outcome partially by driving greater infarct volume growth in acute ischemic stroke with EVT patients.` IL-6, interleukin-6; LOV, large vessel occlusion; EVT, endovascular therapy; CT, computed tomography. The figure was created with BioRender.com.

This study demonstrated that elevated peripheral IL-6 levels were independently associated with unfavorable 90-day outcomes in AIS patients with LVO treated with EVT, with an OR of 1.02 in multivariate analysis. Furthermore, through mediation analysis, we identified that postoperative infarct volume growth partially explained the detrimental effect of IL-6 on clinical outcomes (Graphic abstract, **Figure 7**).

The three core mediation assumptions were verified and satisfied in the present study. First, sequential ignorability was guaranteed by strict temporal causality (EVT treatment, post-operative IL-6 and functional outcome, which ensure irreversible temporal sequence and exclude reverse causality) and comprehensive adjustment for major clinical confounders (The model incorporated all variables that differed between favorable outcome group and unfavorable outcome group). Second, no residual confounding was achieved by excluding patients with systemic inflammation or infection (**Figure 2**) and adjusted by multivariable regression, substantially eliminating baseline imbalance between groups. Third, standardized blood sampling, uniform IL-6 laboratory testing, and blinded functional outcome assessment collectively ruled out measurement bias and enabled consistent, reliable variable quantification.

Numerous clinical studies have shown that IL-6 was associated with poor clinical outcomes in AIS patients after EVT (7, 8), however, a small-scale study also indicated that IL-6 was not independently associated with poor outcomes, defined as mRS score of 5–6 at 3 month (15). Our study confirmed that IL-6 may not only exhibited weak association of unfavorable prognosis, but also first validated infarct volume growth as a mediator of this effect. Increasing evidences from both experimental models (16, 17) and clinical cohorts (18, 19) implicates IL-6 in infarct volume progression, where it may functionally contribute to ischemic injury after reperfusion through mechanisms such as blood-brain barrier disruption, enhanced neutrophil infiltration, or amplification of local neuroinflammation (20, 21). Although no significant differences in inflammatory factors were observed between the two prognostic groups among elderly patients aged ≥85 years in this study, which may be attributable to the small sample size, age-related systemic hypofunction, latent infection, organ failure, as well as immunosenescence and blunted inflammatory responses in ultra-elderly individuals (22–24), Spearman correlation analysis still identified a significant correlation between IL-6 and infarct volume growth. These findings collectively support that IL-6 shows an independent statistical association with clinical outcomes in EVT-treated AIS patients, with infarct expansion serving as at least a partial mediator of this correlative relationship.

The peripheral immune status in AIS also warrants attention. Previous research has demonstrated that AIS can trigger a state of peripheral immunosuppression (25), which may subsequently increase susceptibility to infections and thereby contribute to unfavorable clinical outcomes (26, 27). Serum IL-6 is a well-characterized inflammatory cytokine whose expression dynamics are significantly altered following AIS onset (8, 28, 29). Our study suggests that in addition to affecting outcome by influencing infarct volume growth, IL-6 may also impact outcomes through additional mechanisms, potentially by contributing to post-stroke infections, such as stroke-associated pneumonia.

Tocilizumab, a humanized monoclonal antibody targeting the IL-6 receptor, suppresses IL-6 signaling and the downstream inflammatory cascade. Preclinical studies have demonstrated that tocilizumab could reduce infarct volume and ameliorate severity in rodent models of transient middle cerebral artery occlusion (30–32). The findings of our study only support clinical association rather than direct therapeutic evidence. However, the IRIS (Interleukin-6 Receptor Inhibition in Stroke) trial, a randomized, double-blind, placebo-controlled study, evaluated the efficacy and safety of tocilizumab in patients with AIS due to anterior circulation LVO who were undergoing thrombectomy (33). Notably, results from this trial indicated that tocilizumab tended to attenuate infarct volume growth at 72 hours post-treatment with a favorable safety profile, implying that combined use with EVT could yield greater clinical benefits for AIS patients with LVO (34).

Our study primarily explored the correlations among IL-6, infarct volume expansion and clinical prognosis, future in-depth investigations into the associations between this inflammatory cytokine and other pathological mechanisms are warranted. Previous research has confirmed that white matter lesions are the hallmark pathological feature of sporadic cerebral small vessel disease (35, 36), further studies exploring the relationships between chronically elevated IL-6, progressive white matter alteration rate assessed by image sequence and long-term ischemic stroke prognosis are essential. Furthermore, hemorrhagic transformation represents a well-established adverse prognostic factor for patients with LVO undergoing EVT (37), additional clinical studies are required to clarify whether IL-6 exacerbates hemorrhagic transformation and thereby modulates stroke prognosis. In addition, AIS triggers robust hypothalamic-pituitary-adrenal axis activation, which tightly linked to post-stroke functional outcome (38), It is critical to elucidate the interaction between IL-6 mediated neuroinflammation and the hypothalamic-pituitary-adrenal axis in AIS patients. Collectively, these multi-dimensional research directions will help systematically delineate the integrated pathways whereby IL-6 induces cerebral injuries in AIS and chronic cerebral small vessel disease.

Several limitations of this study must be acknowledged. First, the single time-point measurement of inflammatory factors within 24 hours may not capture the dynamic evolution of the inflammatory response. In particular, we only measured IL-6 within 24 hours after EVT without collecting pre-EVT serum IL-6 samples, which we cannot fully distinguish whether elevated IL-6 post-procedural drives unfavorable outcome or simply reflects the severity of cerebral ischemic injury and reperfusion triggered inflammatory responses. Further prospective studies with longitudinal IL-6 measurements obtained both before and after EVT are warranted to distinguish inflammatory responses stemming from primary ischemic injury or reperfusion induced secondary neuroinflammation. Second, although both baseline MRI and follow-up CT are clinically validated for infarct volume measurement, the use of different imaging modalities for assessment could introduce methodological discrepancies and potential inaccuracies to infarct volume calculation. Third, given the retrospective study design, our findings only demonstrate statistical correlations rather than definitive causal relationships, further prospective clinical studies are needed to verify this mediating relationship, while fundamental research is necessary to draw more rigorous causal inferences. Forth, relatively modest sample size may compromise the robustness and reproducibility of our mediation analysis. Given the limited sample, additional stratified or covariate-adjusted sensitivity analyses would lead to excessive subdivision of the dataset and further reduce statistical power, which could generate unreliable unstable estimates. Instead, we have adjusted core confounders in the mediation models. Notably, we excluded the patients who died within 7 days, which may introduce selection bias and potentially underestimate the association between inflammatory and clinical outcome. Large prospective cohorts with flexible imaging protocols to gather comprehensive data on critically ill patients will allow accurate quantification of the association between inflammatory burden and poor clinical outcomes. Fifth, nearly half of initially enrolled participants were excluded from the final analysis, which may introduce selection bias. Patients with posterior circulation infarction, without MRI evaluation before EVT, with incomplete follow-up data were excluded from the study. Accordingly, our conclusions cannot be fully generalized to all consecutive LVO AIS patients receiving endovascular therapy, and multi-center cohorts with less strict exclusion criteria are required to validate our results. Finally, the correlation between IL-6 and infarct volume growth was weak (Spearman r = 0.28), which only provides modest evidence to the proposed biological mechanism. Alternatively, the modest correlation observed is reasonable, as infarct volume growth after stroke is driven by several overlapping pathological mechanisms, including excitotoxicity, blood-brain barrier disruption, and various inflammatory cytokines beyond IL-6. Further multi-inflammatory factor studies are warranted to more comprehensively validate this biological pathway.

## Conclusion

IL-6 was associated with unfavorable clinical outcomes after EVT, with this effect being partially mediated by infarct volume growth.

## Data Availability

The original contributions presented in the study are included in the article/**Supplementary Material**. Further inquiries can be directed to the corresponding authors.

## Supplementary material

Supplementary Figure 1 Expression of serum inflammatory factors (IL-2, IL-4, IL-6, IL-10, TNF-α, and IFN-γ) in the favorable versus unfavorable outcome groups among admitted patients with aged 85 years old or more (n=7 vs. n=3), as detected by Navios flow cytometry (A–F). Panel G shows the comparison of infarct volume growth between the two groups in admitted patients with aged 85 years old or more. Panel H shows the Spearman correlation analysis of IL-6 and infarct volume growth in admitted patients with aged 85 years old or more. IL, interleukin; TNF-α, tumor necrosis factor-alpha; IFN-γ, interferon-gamma.
