# Supplementary material for: Serum IL-6 and functional outcome after endovascular therapy: the mediating role of infarct volume growth in acute ischemic stroke
Source: Front Immunol. 2026 Aug 10;17:1800891. doi: 10.3389/fimmu.2026.1800891 (PMC13500271; doi:10.3389/fimmu.2026.1800891)
Supplement: Supplementary file 1 [file Table1.docx]

Supplementary table. Baseline Characteristics of Eligible Patients (Aged 85 Years or More) Stratified by Outcome

|  | **Favorable outcome^#^**  **(n=7)** | | **Unfavorable outcome^#^**  **(n=3)** | ***P*** |
| --- | --- | --- | --- | --- |
| **Demographic characteristics,** **median (SD), or n (%)** | | | | |
| Age, | 87.14 (1.86) | | 86.33 (1.15) | 0.513 |
| Male | 3 (42.86) | | 0 (0) | 0.475 |
| **Vascular risk factors, n (%)** | | | | |
| Hypertension | 4 (57.14) | | 2 (66.67) | 1.000 |
| Diabetes mellitus | 1 (14.29) | | 0 (0) | 1.000 |
| Previous stroke | 2 (28.57) | | 0 (0) | 1.000 |
| Atrial fibrillation | 1 (14.29) | | 2(66.67) | 0.183 |
| Valvular heart disease | 0 (0) | | 0 (0) |  |
| CAD | 2 (28.57) | | 0 (0) | 1.000 |
| Drinking history | 1 (14.29) | | 1 (33.33) | 1.000 |
| Smoking history | 0 (0) | | 0 (0) | 1.000 |
| Heart failure | 0 (0) | | 0 (0) |  |
| Cerebral hemorrhage | 0 (0) | | 0 (0) |  |
| **TOAST classification, n (%)** 0.167 | | | | |
| LA | 5 (71.43) | | 0 (0) |  |
| CE | 2 (28.57) | | 3 (100) |  |
| Others^*^ | 0 (0) | | 0 (0) |  |
| **Occlusion location, n (%)** |  | |  | 1.000 |
| ICA | 3 (42.86) | | 1 (33.33) |  |
| MCA | 4 (57.14) | | 2(66.67) |  |
| TO | 0 (0) | | 0 (0) |  |
| **mTICI** **scale , n (%)** |  | |  | 0.116 |
| 2a | 0 (0) | | 0 (0) |  |
| 2b | 1 (14.29) | | 2 (66.67) |  |
| 3 | 6 (85.71) | | 1 (33.33) |  |
| **Clinical assessment and treatment, median (IQR), mean (SD), or n (%)** | | | | |
| DPT, minutes | 114.43 (40.24) | | 84.33 (48.75) | 0.335 |
| DRT, minutes | 161.00 (142.00,189.00) | | 175.00 (148.00,333.00) | 0.569 |
| Within 4.5-hour time window^&^ | | 2 (28.57) | 1 (33.33) | 1.000 |
| mRS on admission | 0 (0, 0) | | 0 (0, 2) | 0.513 |
| NIHSS on admission | 13.43 (2.99) | | 14.33 (2.52) | 0.661 |
| ASPECT score | 9 (0.82) | | 9.33(0.58) | 0.545 |
| Intravenous thrombolysis | 3 (42.86) | | 1 (33.33) | 1.000 |
| Baseline infarct volume (ml) | 2.69 (2.65) | | 37.89 (64.38) | 0.153 |
| FIV (ml) | 6.17 (0.00, 10.34) | | 90.00 (2.18, 135.47) | 0.207 |
| Infarct Volume Growth (ml) | 2.17 (-4.00,10.34) | | 0.74 (-22.22, 135.47) | 0.732 |
| Hemorrhagic transformation | 0 (0) | | 1 (33.33) | 0.300 |
| **Laboratory parameters (mmol/l), median (IQR), or mean (SD)** | | | | |
| Fasting Blood Glucose | 5.98 (0.95) | | 6.52 (1.00) | 0.443 |
| TG | 0.84 (0.34) | | 0.88 (0.37) | 0.869 |
| LDL-C | 2.39 (0.98) | | 2.40 (1.06) | 0.980 |
| HDL-C | 1.36 (1.24, 1.43) | | 1.02 (0.92, 1.83) | 0.425 |
| **Inflammatory factor (pg/mL), median (IQR), or mean (SD)** | | | | |
| TNF-α | 2.44 (1.34) | | 1.42 (0.70) | 0.259 |
| IFN-γ | 3.97 (1.36) | | 3.23 (2.34) | 0.536 |
| IL-10 | 4.90 (3.59) | | 4.85 (1.69) | 0.984 |
| IL-2 | 3.60 (1.11) | | 2.71 (0.96) | 0.266 |
| IL-4 | 2.50 (0.98) | | 1.31 (0.38) | 0.082 |
| IL-6 | 22.16 (12.38) | | 22.69 (21.98) | 0.961 |

Abbreviations: Interquartile range, IQR; CAD, Coronary Artery Disease; TOAST, Trial of Org 10172 in Acute Stroke Treatment; mTICI, modified Thrombolysis in Cerebral Infarction scale; LA, large-artery atherosclerosis; CE, cardio-embolism; ICA, internal carotid artery; MCA, middle cerebral artery; TO, tandem occlusions; DPT, door-to-puncture time; DRT, door-to-reperfusion time; ASPECT, Alberta Stroke Program Early Computed Tomography Score. The 4.5-hour time window, The 4.5-hour thrombolysis time window; NIHSS, National Institutes of Health Stroke Scale; mRS, Modified Rankin Scale; TG, triglycerides; LDL-C, low-density lipoprotein cholesterol; HDL-C, high-density lipoprotein cholesterol; FIV, final infarct volume; TNF-α, tumour necrosis factor-α; IFN-γ, interferon-γ; IL, interleukin.

^*^Others include small-vessel occlusion, stroke of other determined etiology and undetermined etiology.

^#^Favorable outcome was defined as a mRS score of 0-2 at the 90-day follow-up, and mRS score of 3-5 was considered as Unfavorable outcome.

^&^The 4.5-hour time window refers to onset-to-arrival time ≤4.5 h at our center, several patients presented beyond this window yet received pre-transfer thrombolysis at outside hospitals.
